# Community-level determinants of obesity: harnessing the power of electronic health records for retrospective data analysis

**DOI:** 10.1186/1472-6947-14-36

**Published:** 2014-05-08

**Authors:** Caryn Roth, Randi E Foraker, Philip RO Payne, Peter J Embi

**Affiliations:** 1 Department of Biomedical Informatics, College of Medicine, 250 Lincoln Tower 1800 Cannon Drive, 43210 Columbus, OH, USA; 2 Division of Epidemiology, College of Public Health; The Ohio State University, Columbus, OH, USA

**Keywords:** Electronic health record, Obesity, Data integration, Community data, Clinical research informatics, Prevention, Access, Social determinants of health

## Abstract

**Background:**

Obesity and overweight are multifactorial diseases that affect two thirds of Americans, lead to numerous health conditions and deeply strain our healthcare system. With the increasing prevalence and dangers associated with higher body weight, there is great impetus to focus on public health strategies to prevent or curb the disease. Electronic health records (EHRs) are a powerful source for retrospective health data, but they lack important community-level information known to be associated with obesity. We explored linking EHR and community data to study factors associated with overweight and obesity in a systematic and rigorous way.

**Methods:**

We augmented EHR-derived data on 62,701 patients with zip code-level socioeconomic and obesogenic data. Using a multinomial logistic regression model, we estimated odds ratios and 95% confidence intervals (OR, 95% CI) for community-level factors associated with overweight and obese body mass index (BMI), accounting for the clustering of patients within zip codes.

**Results:**

33, 31 and 35 percent of individuals had BMIs corresponding to normal, overweight and obese, respectively. Models adjusted for age, race and gender showed more farmers’ markets/1,000 people (0.19, 0.10-0.36), more grocery stores/1,000 people (0.58, 0.36-0.93) and a 10% increase in percentage of college graduates (0.80, 0.77-0.84) were associated with lower odds of obesity. The same factors yielded odds ratios of smaller magnitudes for overweight. Our results also indicate that larger grocery stores may be inversely associated with obesity.

**Conclusions:**

Integrating community data into the EHR maximizes the potential of secondary use of EHR data to study and impact obesity prevention and other significant public health issues.

## Background

The prevalence of obesity and overweight in the United States (US) has drastically increased over the past 20 years, with over one third of adult men and women considered obese in 2010. An additional third of the adult population is overweight, and approximately 18% of children and adolescents aged 6–19 are obese [1,2]. Beyond links with higher all-cause mortality [3], obesity is associated with numerous comorbidities, including hypertension, type II diabetes, coronary heart disease, stroke, osteoarthritis, and cancers [4]. Obesity-related diseases cost $19.1 billion annually in the US alone [5], heavily straining the health care system and further demonstrating the need to focus on treatment and prevention of this dangerous and costly disease [6].

While the causes of obesity are multifactorial, environmental factors may influence the development of the disease [7,8]. Through Geographic Information Systems (GIS) and other spatial analysis methods, researchers can map and explore the link between area of residence and health outcomes like obesity and overweight [9,10]. By characterizing neighborhoods according to community-level factors, we may be able to study their effects on disease distributions, including obesity [11-13]. For example, environmental factors that promote physical activity such as parks, walkable neighborhoods and recreational facilities have been associated with lower rates of obesity [14-16]. Similarly, increased access to fruits and vegetables through neighborhood grocery stores and farmers’ markets has also been associated with lower rates of obesity [16-18], even though the selection of healthy food at these stores varies by size and type of store [19]. Socioeconomic indicators like education and income, which are often intricately intertwined with place of residence, have been linked to obesity as well [20-22]. Understanding which of these underlying environmental factors contribute most to overweight and obesity is crucial for determining prevention targets.

In parallel to the rising obesity epidemic, the use of electronic health records (EHRs) in the US has also skyrocketed. This increase is due in a large part to the 2009 passage of the Health Information Technology for Economic and Clinical Health (HITECH) Act, part of the American Recovery and Reinvestment Act (ARRA), a federal stimulus bill which provides incentive payments for health care providers to offset costs associated with EHR implementation [23]. With the passage of HITECH, the US Congressional Budget Office estimated that by 2019, 70% of hospitals and 90% of physicians would adopt EHRs for their practices [24]. Most of the stimulus bill funds are allocated as Centers for Medicare and Medicaid Services incentive payments for “meaningful use” of health information technology, mandating that EHRs contribute to enhanced quality, efficiency and engagement in healthcare [25,26].

Beyond using the EHR to enhance patient care, the increase in adoption and emphasis on meaningful use presents significant opportunity to utilize EHRs for secondary research purposes [27]. As these data are already collected, the research costs lie only in the analysis, and patient-level clinical data can be aggregated for research that impacts the larger population [28,29]. While data extraction, workflow and regulatory barriers surrounding the reuse of EHR data still exist, EHRs may ultimately allow scientists to surmount translational research barriers by capitalizing on these rich data sources, fully enabling patient-centered outcomes and comparative effectiveness research [30,31].

As secondary use of EHR data becomes more sophisticated, there is a need to expand the way we think about and leverage these data. In the case of obesity prevention, previous studies linking environmental factors are varied in their methods, subjects and results. If we can harness the tremendous data source already available in the EHR, we will be able to study these factors in a more systematic and powerful way. However, since information on key community-level factors is not contained in the EHR, current studies on obesity in the context of *only* the EHR are limited. As we try to shift the focus of healthcare towards wellness and prevention, these community data that exist beyond the confines of clinics and hospitals are increasingly important [32,33]. We hypothesized that adding community-level data on socioeconomic and obesogenic environmental factors would enrich EHR-derived data and enable us to better study overweight and obesity in a patient population. To explore this methodology as a way of generating future hypotheses concerning obesity-related factors, we specifically focused on community-level factors previously correlated with obesity, such as access to nutritious food and physical activity, as well as income, population size and education. Our study aimed to investigate possible associations between these community-level factors and the prevalence of overweight and obesity, in order to generate specific hypotheses and demonstrate the validity of the secondary use of these data sources.

## Methods

### Study design

We obtained patient-level data from The Ohio State University Wexner Medical Center (OSUWMC), a large academic medical center in Franklin County, Ohio serving a socioeconomically and demographically diverse population. Our 62,701-person final cohort included inpatients and outpatients aged 18–67 with an address of record in Franklin County seen between October 2010 and September 2011. We excluded 24 patients for whom either height or weight was not recorded, and excluded patients whose calculated values for body mass index (BMI, kg/m^2^) were implausible or too extreme. In total, these comprised 1,396 observations where BMI was below 18.5 and 3,417 observations where BMI was above 45. We also removed 4,796 observations where the individual’s race was listed as missing or was from a category that was too small to meet the sample size assumptions of logistic regression. Finally, we excluded 135 patients for whom we did not have an adequate zip code of residence, due to differences in how zip codes that overlap with county lines are classified by our various data sources. The Ohio State University Institutional Review Board approved this research.

### Data sources

As the community-level data of interest for this study was not present in the EHR, we linked diverse data sources for this analysis. From the OSUWMC Information Warehouse, we requested height and weight data from the most recent visit where these data were recorded, as well as gender, race, year of birth and zip code.

We used a database of consumer behavior trends (Nielsen PrimeLocation) to capture socioeconomic and population characteristics for each zip code. These data are collected through phone, mail and online surveys, as well as increasingly through automated methods such as barcode scanners and smartphone applications. We linked socioeconomic data, such as median household income, percent unemployment, percent below poverty level, population size and education with its corresponding zip code.

We grouped education variables into larger categories to reflect categorizations of the US census. For example, we created the variable for percent of population with a high school degree by summing the number of individuals over 25 in each zip code who were high school graduates, the number who had completed some college but had not attained a degree and the number who had an associate’s degree. We divided this sum by the total number of individuals in each zip code and then multiplied this value by 100. Similarly, percent of population with a college degree encompassed those over 25 who attained a bachelor’s degree, master’s degree, professional school degree or doctorate degree, divided by the total number of individuals in each zip code and multiplied by 100.

**Figure 1:**
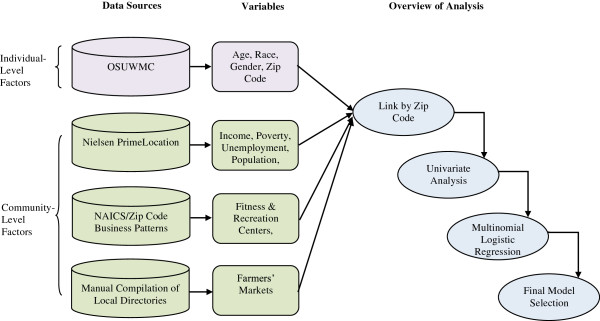
**Data sources, variables and workflow overview.** Patient-level data from the OSUWMC was linked by zip code to area-level data describing socioeconomic and obesogenic factors. Multinomial logistic regression was used to construct a final model describing associations between these community-level factors and overweight and obesity.

We quantified the number of recreational centers and grocery stores in each zip code by examining North American Industry Classification System (NAICS) data codes 713940 for fitness and recreational sports centers and 445110 for supermarkets and other grocery stores, excluding convenience stores [34]. We looked these codes up in Zip Code Business Patterns Database, maintained by the US Census, to find the number of each type of business in each zip code [35]. We also enumerated farmers’ markets in each zip code by manually compiling various online and local directories and confirming that each of these markets was still active with a phone call or visit. We included markets that occurred at least once every two weeks and ran for at least three consecutive months. We normalized the number of recreation centers, grocery stores and farmers’ markets in each zip code by dividing by the population in that zip code and multiplying by 1,000. Data sources, potential variables of interest and an overview of the analysis steps are shown in Figure 1.

### Analysis

Our primary outcome was BMI category. We grouped patients according to established cutoffs for normal weight (18.5 ≤ BMI < 25), overweight (25 ≤ BMI < 30) and obese (30 ≤ BMI < 45).

We performed multinomial logistic regression to estimate odds ratios and 95% confidence intervals (OR, 95% CI) comparing overweight and obese, respectively, to normal weight. We clustered the patients in the analysis at the level of the zip code. We assessed all variables for collinearity and removed median household income, average household income, median household effective buying power and average effective buying power from the analysis due to high collinearity with per capita income. We screened each remaining variable for an association with BMI at the α = 0.1 level. Next we used backwards elimination to examine the significance of each variable in the model at the α = 0.05 level, and then allowed each variable eliminated at previous steps a chance to re-enter the final model [36]. We assessed for linearity in the logit using fractional polynomial model comparisons, and accounted for interactions between variables. We used Stata (Statacorps, SE version 12) for this analysis.

After creating our final model, we more deeply explored some of the factors that we hypothesized might have more variability within and between zip codes. We plotted grocery stores in the county on a map shaded with the percentage of obese individuals from our cohort in each zip code, and examined larger stores separately to visually assess whether the size of grocery store might be associated with BMI.

## Results

Table 1 shows the overall distributions of age, gender and race for our full final patient cohort, as well as broken down by BMI category. The overall mean age was 44, 62% of our subjects were female, and 72%, 25%, and 3% of the sample were Caucasian, African American, and Asian, respectively. Of these, 20,779 (33%) were normal weight, 19,567 (31%) were overweight and 22,355 (36%) were obese.

**Table 1:** Characteristics of patient population

	**Full cohort**	**Normal weight**	**Overweight**	**Obese**
**Population (count)**	62,701	20,779	19,567	22,355
**Age (mean, standard deviation)**	43.68, 13.31	40.37, 13.70	44.58, 13.12	45.95, 12.46
**Female (%)**	62.04	67.50	53.97	64.03
**Race**				
**Caucasian (%)**	71.75	76.49	73.38	65.91
**African American (%)**	25.03	17.79	23.48	33.10
**Asian (%)**	3.23	5.72	3.14	0.99

**Figure 2:**
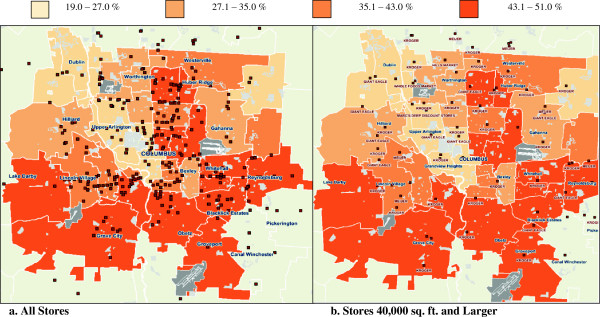
**Percent obese by zip code, 2010, with grocery stores plotted. a** depicts all NAICS-classified grocery stores, whereas **b** only includes grocery stores larger than 40,000 square feet. Fewer large grocery stores are observed in the areas with the highest levels of obesity.

Mean BMI varied across the zip codes from 25.0 to 30.7. The zip code with the lowest average BMI surrounds the large state university, and thus is home to many students and younger, often healthier individuals. Yet even there, the average BMI (25.0) is exactly at the cutoff of overweight, demonstrating the high prevalence of overweight and obese across our population. Figure 2 depicts the percentage of obese individuals in each zip code.

When adjusted for age, race and gender, we found that more farmers’ markets/1,000 people [0.19, (0.10-0.36)], more grocery stores/1,000 people [0.58, (0.36-0.93)] and higher percentage of college graduates/10% increase [0.80, (0.77-0.84)] were all significantly associated with lower odds of obesity. The same factors showed a protective effect for overweight as well, exhibiting a dose response relationship with the magnitude of the ORs (Table 2).

**Table 2:** Multinomial logistic regression, adjusted for age, race, gender (ORs, 95% CI)

	**Overweight**	**Obese**
**College-educated per 10% increase**	0.94 [0.92, 0.97]	0.80 [0.77, 0.84]
**Farmers’ markets per 1,000 people**	0.42 [0.32, 0.55]	0.19 [0.10, 0.36]
**Grocery Stores per 1,000 people**	0.74 [0.57, 0.98]	0.58 [0.36, 0.93]

Our results indicate that for every 10% increase in percent of population with a college education, the odds of being overweight decreased by 6% and the odds of being obese decreased by 20%, holding age, race, gender, farmers’ markets and grocery stores constant. Similarly, for each additional farmers’ market per 1,000 people, the odds of being overweight decreased by 58% and the odds of being obese decreased by 81%, holding age, race, gender, grocery stores and percent with a college degree constant. Finally, for every additional grocery store per 1,000 people, the odds of being overweight decrease by 26% and the odds of being obese decrease by 42%, holding age, race, gender, farmers’ markets and percent with a college degree constant.

The size of grocery stores varied widely across the zip codes in our catchment area. Figure 2a depicts all NAICS-classified grocery stores, whereas Figure 2b includes grocery stores larger than 40,000 square feet. We observe fewer large grocery stores in zip codes with higher rates of obesity.

## Discussion

More farmers’ markets, grocery stores, and higher percentage of college-educated residents are associated with lower odds of overweight and even lower odds of obesity. These results are consistent with many studies that found similar associations between obesity and farmers’ markets, grocery stores, and college education using different study designs [15-18,22]. Contrary conclusions from the literature generally examined obesity in a different context, for example in rural areas [20]. Like our results, another study found that greater access to grocery stores was associated with lower rates of obesity in urban areas, but the same study found higher obesity rates in rural areas with more grocery store access [37]. Clearly the verdict is still out on the exact mechanism by which community-level factors impact obesity, which likely is due to the multifactorial nature of the disease. Thus it will be crucial to focus on strategies that are context- and area-specific. An analysis that incorporates environmental factors is necessary to get to the root of the problem and find solutions to obesity and overweight.

While secondary use of EHRs has become a major focus of clinical research informatics work, [29,38] to our knowledge, none of these studies have attempted to look at associations between community-level factors and overweight and obesity. The data used in our work are currently not available in the EHR; however, bringing in additional data is not a new concept. Many are working towards integrating genomic data into the EHR to enhance information for clinicians at the point-of-care or for research and discovery purposes [39-42]. Our results indicate that this type of integration may provide value for community- and area-level data as well. Rather than collecting the datasets separately and integrating them only for our analysis, these data could be made available at the point-of-care, enabling clinicians to better factor a patient’s environment into health recommendations and treatments and researchers to more readily perform this type of analysis.

Studying the impact of community-level data on health in this way also raises additional questions as we explore public health implications for this type of work. For example, while we treated all grocery stores in the NAICS classification system as equivalent for this analysis, in fact we know that grocery stores do not all provide equal access to healthy food. Larger grocery stores often have more ability to buy in bulk and provide discounts on produce, leading to more affordable and accessible healthy foods. On the other hand, smaller grocery stores are often limited in fresh produce and offer more packaged foods that are non-perishable. These options contain more fat, sugar and salt than fresh foods, and thus those who shop at these smaller stores have more limited healthy options. When we compared all grocery stores in Franklin County to just the stores 40,000 square feet or larger, we observed that the distribution of stores differed across zip codes in the catchment area. This may explain the wider CIs as estimated by the regression model, as areas on the map with higher obesity rates generally correspond to those with fewer large grocery stores. In addition, the classification of variables in this analysis may have had a huge impact on its results, and we cannot ignore these implications as we think about using these results in the future to affect change. For example, if we were to add a grocery store to a zip code with a high prevalence of obesity and evaluate its effect on obesity rates in a longitudinal manner, this work suggests that the size and type of store would matter.

Augmenting our EHR data proved to be a useful and valid method for hypothesis generation, a methodology which we will continue to use with new community-level factors and larger population areas. This case study demonstrates the potential of combining community-level and clinical data for studying overweight and obesity, among other population health issues. We started with factors that we already believed would be associated with obesity, but this type of analysis can also incorporate variables where the associations are not established or even yet-hypothesized. Moreover, without any changes to existing EHR systems, zip code data are available for research purposes. By adding data about the socioeconomic and obesogenic factors in an individual’s environment, we can paint a fuller picture of a patient population and gain better insight into the clinical and non-clinical factors that contribute to overweight and obesity. Understanding the diverse environments of our patients can enhance comparative effectiveness research and provide insight into why certain treatments or interventions are more effective for specific populations. In this way, we can also better predict the outcomes for individual patients, promoting a more robust learning health system that incorporates a holistic representation of each patient.

### Challenges, limitations and future directions

While promising, this work also reveals significant obstacles for leveraging data collected in today’s healthcare systems to fully enable patient-centered outcomes research. We used data from the EHR, which can be biased or faulty due to data entry errors. We did take this into account and removed records with implausible values for BMI in our analysis. In addition to the data quality issue, we removed these participants with the assumption that they were more likely to be underweight or extremely overweight due to illness, and could disproportionately represent the sicker inpatients in our medical center. These decisions, as well as the fact that this study explored health and community factors in one Midwestern region, may limit the generalizability of our results to other states or populations.

Beyond erroneous values, EHR-derived data present additional challenges when used for secondary purposes such as research. These issues include missing data, heterogeneous data entry practices over time and between physicians, and data stored in text fields, which are difficult to access and use for large-scale research [30]. Even data stored in an easily-accessible structured format may be unreliable or unusable for research, due to its initial intended purpose, for example billing [43]. Furthermore, unknown provenance and insufficient granularity may limit the data from answering the research question of interest [44]. Many are working to address such issues, for example tackling the free text issue by developing sophisticated Natural Language Processing (NLP) techniques [45,46]. As the larger informatics community works to address such obstacles in the secondary use of EHR data, we can still utilize this rich data source, keeping cognizant of its limitations.

While more farmers’ markets may cause lower rates of overweight and obesity, they might also be an indicator of a health-conscious community that is more likely to advocate for farmers’ markets in the neighborhood. Without further longitudinal study, we cannot verify the direction of causation. Similarly, the nature of this cross-sectional study means we are looking at a snapshot in time. Obesity and overweight do not develop overnight; neither do robust farmers’ markets. From this analysis we can only infer that the two are associated, but without tracking the change in farmers’ markets or the movement of individuals between different communities, we cannot account for the temporality of the observed association.

We also hypothesize that using community-level data aggregated at the zip-code level rather than at a more granular, but difficult to obtain, smaller unit of localization may misclassify patients in terms of area-level health factors. As zip codes are designed to optimize mail delivery, they are not homogenous and therefore cannot fully represent the obesogenic or socioeconomic environments of their inhabitants [47]. To address this issue, we are working to categorize our patients into smaller groupings such as census tract, which would allow us to more fully explore variation of community-level characteristics within zip code. However, obtaining the patient address geocoded to the level of the census tract for such a large cohort is a challenging endeavor, given the limitations of current data warehouse processes and the time involved.

Additional challenges arise from linking data from multiple sources. While some data will match according to the linker in each dataset, other entries will remain unmatched [48]. We were confronted with this issue when we queried the PrimeLocation database for community-level data describing zip codes within Franklin County, which did not in fact cover all the zip codes that comprised our patient cohort. This mismatch was due to the fact that zip codes and counties do not share perfect overlap, so the PrimeLocation classification system did not include some of the zip codes which were not fully contained in the county borders. This forced us to exclude patients residing in those districts from our analysis, which may have induced a selection bias. Furthermore, each distinct data source contained data from one point in time, but these do not necessarily match. For example in our analysis, our patient data were from 2010–2011, and the PrimeLocation data were from 2011. However, we could not get 2011 data on number of establishments from the US Census, so we had to settle for older data, adding possible misclassification into the analysis. Further integration challenges likely would have arisen had we been able to acquire this type of socioeconomic and community resource data at the more granular level of the individual patient.

Our approach for combining and analyzing these data was semi-manual, but we are exploring options for better integrating and sustaining these data sources long-term. For example, by incorporating community-level data into EHRs directly, we would not only attain more granular data at the patient level, but we would be better able to validate and analyze it in a longitudinal fashion. Physicians could use this additional patient context to make more relevant health recommendations, which might improve compliance and outcomes. Unfortunately, adding external data into the EHR presents its own set of challenges related to accuracy and timeliness, which may not make this option viable on a large-scale. However, efforts to integrate community-level data into enterprise data warehouses are underway, and these novel methods present a promising approach to systematically integrate these data to answer such research questions [49]. Our current work makes the case for the importance of such efforts and informatics solutions to facilitate this type of research.

The spatial issue impacts individuals who live on or near zip code boundaries and may actually have more access to community-level factors of a neighboring zip code. Since we lacked individual location coordinates for those in our study, we could not account for this phenomenon in our analysis. For the same reason, we could not measure distance to specific community-level resources such as grocery stores, but instead had to use the number of such resources per zip code, standardized by population size, as a proxy. We also lacked information about how far people will travel to purchase food, which others have suggested may be important considerations in actual food purchasing habits [50].

We plan to explore other methodologies, such as those from the data science domain, which might enable us to draw additional insights from this data [51]. We also will explore creating a predictive model with these data, in order to see if we can better predict a patient’s BMI category using community-level data. As we expand this work to other counties, states and health systems, we can continue to test different methodologies that might be appropriate for this type of work.

## Conclusions

We combined EHR-derived patient level data with community-level data to identify factors associated with elevated BMI. Our results demonstrate the benefit of combining these data sources to develop hypotheses about population health across a diverse community. As current changes in care and reimbursement at the national level shift focus from acute care to a wellness-based system, this type of work exploring data-driven determinants of health at a broad level is critical. The secondary use of EHR data facilitates a learning health system where we can effectively utilize the data contained in the EHR for new insight and knowledge. In order to realize its full potential, we must look beyond the EHR for supplemental data sources and integrate community-level factors into studies of chronic diseases. Contextual factors have already proven to be integral to the development of chronic disease, and further study may help illuminate how we can halt the progression of epidemics like obesity and improve health.

## Abbreviations

ARRA: American Recovery and Reinvestment Act; BMI: Body mass index; EHR: Electronic health record; GIS: Geographic Information Systems; HITECH: Health Information Technology for Economic and Clinical Health; NAICS: North American Industry Classification System; NLP: Natural Language Processing; OSUWMC: The Ohio State University Wexner Medical Center; US: United States.

## Competing interests

The authors declare that they have no competing interests.

## Authors’ contributions

CR conceived of the study, collected the data, led the statistical analysis and drafted the manuscript. RF, PP and PE all helped guide the analysis, provided domain knowledge and expertise and helped shape the manuscript. All authors read and approved the final manuscript.

## Pre-publication history

The pre-publication history for this paper can be accessed here:

http://www.biomedcentral.com/1472-6947/14/36/prepub
